# Analysis of population structure and genetic diversity of *Camellia tachangensis* in Guizhou based on SNP markers

**DOI:** 10.1007/s11033-024-09632-0

**Published:** 2024-06-01

**Authors:** Dejun Huang, Suzhen Niu, Dingchen Bai, Zhifei Zhao, Caiyun Li, Xiuling Deng, Yihan Wang

**Affiliations:** 1 Institute of Tea, Guizhou university, Jiaxiu South Road, Guiyang, Guizhou China; 2 Institute of Agro-Bioengineering, Guizhou university, Xueshi Road, Guiyang, Guizhou China

**Keywords:** Tea, *Camellia tachangensis*, Guizhou Plateau, Population structure, SNP

## Abstract

**Background:**

*Camellia tachangensis* F. C. Zhang is a five-compartment species in the ovary of tea group plants, which represents the original germline of early differentiation of some tea group plants.

**Methods and results:**

In this study, we analyzed single-nucleotide polymorphisms (SNPs) at the genome level, constructed a phylogenetic tree, analyzed the genetic diversity, and further investigated the population structure of 100 *C. tachangensis* accessions using the genotyping-by-sequencing (GBS) method. A total of 91,959 high-quality SNPs were obtained. Population structure analysis showed that the 100 *C. tachangensis* accessions clustered into three groups: YQ-1 (Village Group), YQ-2 (Forest Group) and YQ-3 (Transition Group), which was further consistent with the results of phylogenetic analysis and principal component analyses (PCA). In addition, a comparative analysis of the genetic diversity among the three populations (Forest, Village, and Transition Groups) detected the highest genetic diversity in the Transition Group and the highest differentiation between Forest and Village Groups.

**Conclusions:**

*C. tachangensis* plants growing in the forest had different genetic backgrounds from those growing in villages. This study provides a basis for the effective protection and utilization of *C. tachangensis* populations and lays a foundation for future *C. tachangensis* breeding.

**Supplementary Information:**

The online version contains supplementary material available at 10.1007/s11033-024-09632-0.

## Introduction

Tea is one of the three major non-alcoholic beverage crops in the world and has important health, economic, and ecological value. The Guizhou Plateau is one of the core origins of tea plants and has a rich tea germplasm due to its distinct geographical location and suitable ecological environment [1]. The germplasm of wild tea plants includes valuable genetic resources for studying their domestication and breeding. The Theaceae family member *Camellia tachangensis* is a wild tea plant that was discovered and named by Zhang Fangci. *C. tachangensis* is only distributed in the border area of Yunnan, Guangxi, and Guizhou, where it is widely distributed [2–4]. *C. tachangensis* reprents a type of tea tree that has not been domesticated in tea gardens and is mostly wildly grown. The majority of *C. tachangensis* plants grow in forests or around their perimeter, and a small part grows in villages or farmlands, although few are used to make tea for drinking. Some are used to protect terraces or as landscape trees or sacrificial trees in front of graves. Recently, with advances in *C. tachangensis* research, its germplasm has been found to containkey nutritional components that meet various human needs. and display excellent comprehensive properties for the development of its tea industry [4]. The significance and potential of the *C. tachangensi* germplasm can be further elucidated by investigating its origin and evolution. Molecular markers allow the detection of variations or polymorphisms in specific regions of DNA among individuals in a population [5]. Examples include restriction fragment length polymorphisms, random amplified polymorphic DNA (RAPD), amplified fragment length polymorphisms, simple sequence repeats (SSRs), and single-nucleotide polymorphisms (SNPs). SNPs, the most common type of genetic variation, have been widely used in research on natural populations to investigate the genetic diversity and population structure of agricultural and horticultural crops such as maize [6], wheat [7], pepper [8], potatoes [9], apples [10], and tea. For example, Pang et al. (2020) identified 327,609 SNPs among 768 wheat cultivars using genotyping-by-sequencing (GBS). Feng et al. (2020) identified 38,395 SNPs among 112 cultivated and wild peppers at a genome-wide level using the GBS method and provided genetic evidence of multiple species splitting events or multiple lineage splitting events. A total of 112,072 SNPs were analyzed by Zhao et al. (2022), who also performed phylogenetic analysis, PCA and population structure analysis were carried out to explore the genetic diversity and geographical distribution characteristics of cultivated tea trees in Guizhou Plateau. The results showed that there were significant differences between the ancient tea cultivars in the Yangtze River basin and the Pearl River Basin [32]. . They further revealed cluster relationships and verified three inferred populations. Niu et al.(2019) [4], analyzed the population structure of 415 tea accessions with 79,016 SNPs and identified four groups: pure wild type, admixed wild type, ancient landraces, and modern landraces.

GBS, a reduced-representation genome sequencing technique, can be used to identify SNPs to perform genotyping studies for germplasm identification and analysis [11], genetic linkage map construction [12], genome-wide association analysis [13], and molecular marker-assisted breeding [14]. For example, Babu et al. (2020) used GBS to conduct a genome-wide association study (GWAS) that identified candidate genes for traits related to yield and oil-yield in palm. Kaur et al. (2021) used GBS to develop a genetic linkage map of *Momordica charantia* L. containing 3,144 SNP markers, 15 linkage groups, and spanning 2,415.2 cM with an average marker distance of 0.7 cM. However, the genetic diversity and population structure analysis for *C. tachangensis* population have not yet been reported. In our previous research [4], *C. tachangensis* was found to cluster into two different groups, although the reason for this phenomenon remained unclear.

In this study, 100 samples of *C. tachangensis* were selected for genetic, phylogenetic and population structure analysis using GBS sequencing technology. 100 samples of *C. tachangensis* were clustered into 3 inferred populations: village group (YQ-1), forest group (YQ-2) and transition group (YQ-3), and the heritage distance of the 3 populations was analyzed. The results showed that the genetic diversity of village group was lowest, that of transition group was highest, and that of forest group was medium. This study provides a theoretical basis for elucidating the population structure of *C. tachangensis* in Guizhou Province and further protecting and utilizing *C. tachangensis*. The present research will lay a foundation for future *C. tachangensis* variety breeding and molecular marker development.

## Materials and methods

### Plant materials

**Fig. 1:**
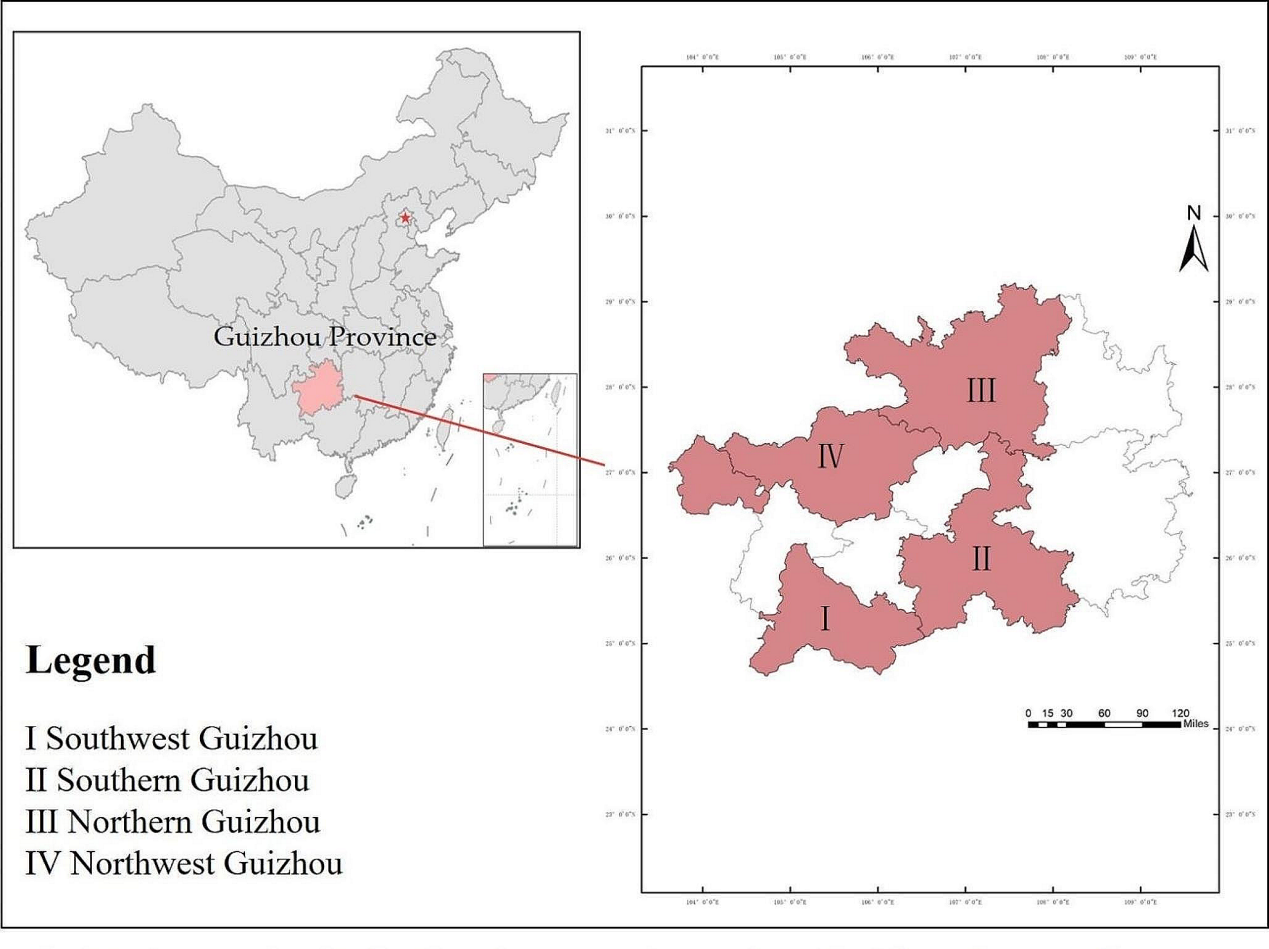
Geographie distribution of tea accession analyzed in this study according to thecollection

The study used 100 *C. tachangensis* accessions based on Niu’s method [4]. Base on the growth location of *C. tachangensis*, the 100 *C. tachangensis* accessions were divided into two types: village population (32 accessions in village and 6 accessions in farmland) and forest population (27 accessions near forest and 35 accessions in forest). The 100 *C. tachangensis* accessions comprised five accessions from Northwest Guizhou, 35 accessions from Southern Guizhou, 54 accessions from Southwest Guizhou and 6 accessions from Northern Guizhou (Fig. 1 Table S1, S2).

### DNA extraction, library construction, and sequencing

We used the Plant genomic DNA Rapid Extraction kit (Biomed Gene Technology) to isolate genomic DNA from samples. DNA integrity was detected on 1% agarose gel, and DNA purity was detected and quantified using Qubit Fluorometer (Invitrogen). In a reaction system of 25µL, we digested 100 NGDNA with 5U SacI and MseI(NEB) and 1 times the restriction enzyme buffer. After digestion, SacAD and MseAD splicers were attached to the digested DNA fragments. Twelve samples were mixed in equal volume and purified using the QIAick PCR purification kit (Qiagen). The purified DNA fragments were then amplified using a mixture of a PCR substrate cocktail and a PCR parent. Amplified fragments of 500–550 bp (including 120 bp aptamers) were obtained by 2% agarose Gel electrophoresis and purified using the QIAick Gel EXTRACTION Kit. The average length of DNA fragments was determined using Agilent DNA 12,000 kit and 2100 BioAnalyzer System(Agilent), and the resulting DNA libraries were quantified using TaqMan probe real-time fluorescent polymerase chain reaction. The paired terminal 150(PE150) sequencing strategy was used on the Illumina HiSeq X Ten platform [4, 15]. 

### Sequence alignment and SNP mining

We have demultiplexed the raw DNA sequence reads based on the barcodes, and trimmed the adapters with a custom Perl script. Any read with a poor quality run of 5 was eliminated. A BWA-MEM (v. 0.7.10)( https://sourceforge.net/projects/bio-bwa/files/) was used to map the reads to the reference tea plant (*Camellia sinensis var. sinensis*) genome [16] (tpdb.shengxin.ren) with its default parameters (Xia et al.2020). According to Niu’study [4]. SNPs were filtered according to several criteria. (1) Biallelic SNPs were selected. (2) “QUAL < 50.0 || QD < 2.0 || FS > 60.0 || MQ < 40.0 || Mapping Quality Rank Sum < -12.5 || Read Pos Rank Sum < -8.0” were used in GATK v. 3.7.0 (https://github.com/broadinstitute/gatk/releases) to filter the SNPs (Mckenna et al.2010). (3) VCFtools (v.0.1.160) (https://github.com/vcftools/vcftools) conserved SNPs with minor allele frequency (MAF) > 0.05 and missing data rates < 20% [17]. The SNP density plot was drawn in CMplot (v.3.7.0) (https://rdrr.io/cran/CMplot/) [18]. The analysis was conducted on 91,959 SNPs from 100 tea accessions.

### Population structure and linkage disequilibrium (LD)

We calculated LD (linkage disequilibrium) using the correlation coefficients (r2) for pairwise SNPs across the genome using PopLDdecay. (v. 3.29) (https://github.com/BGI-shenzhen/PopLDdecay) with its default parameters. A VCF file was converted to a ped file with the assistance of VCFtools [17]. Population structure analysis was performed using ADMIXTURE (v1.3.0) (http://dalexander.github.io/admixture/download.html) software and applying the Bayesian model-based clustering method with maximum likelihood estimation. The number of clusters (K value) were assessed by determining cross-validation errors (CV errors) which were tested from 1 to 9, running ten iterations for each K value. The optimal K value was identified according to the lowest CV error [19]. To distinguish between pure and admixture subgroups, the membership coefficient was set at 0.8 [18]. PCA was performed in TASSEL (v. 5.2.72) (https://tassel.bitbucket.io) [20]. In MEGA (v. 10.2.4) (https://www.megasoftware.net/dload_win_gui), a Neighbor-Joining tree was constructed [21]. The tree was visualized and colored using iTOL (https://itol.embl.de/) [22] .

### Genetic diversity

The inbreeding coefficient (Fis), observed heterozygosity (Ho) and MAF of each inferred population were calculated using Plink v. 1.90 (https://www.cog-genomics.org/plink2/) [23]. VCFtools was used to determine Tajima’s D, nucleotide diversity (Pi), genetic differentiation coefficient (Fst), and Tajima’s D for each inferred population. The formula Nm =(1-Fst) / 4Fst was used to compute Nm (gene flow) [23]. The pairwise inferred populations’ GD was computed using MEGA. In SPSS version 25, significant variations between these indices were found (IBM Corp., Armonk, NY, USA) [24] .

## Results

### Mining SNPs data of *C. tachangensis* germplasm from Guizhou Plateau

A total of 29,393,327 SNP markers were obtained through the GBS of 100 *C. tachangensis* accessions from the Guizhou Plateau. After filtering and screening, a total of 91,959 high-quality SNP markers were used for further analysis. Among the 15 chromosomes, Chr1 had the highest number (7,900) of SNPs and Chr15 had the lowest (3,993). The greatest average SNP density was 35.5 SNPs per 1 Mb on Chr1 and the smallest was 30.5 SNPs per 1 Mb on Chr12. (Fig. S1, Table S3) Transitions accounted for 78.38% (AG 39.17%, CT 39.21%) of SNPs, and transversions accounted for 21.62% (AT 6.76%, AC 5.39%, CG 3.89%, GT 5.58%) (Table 1).

**Fig. 2:**
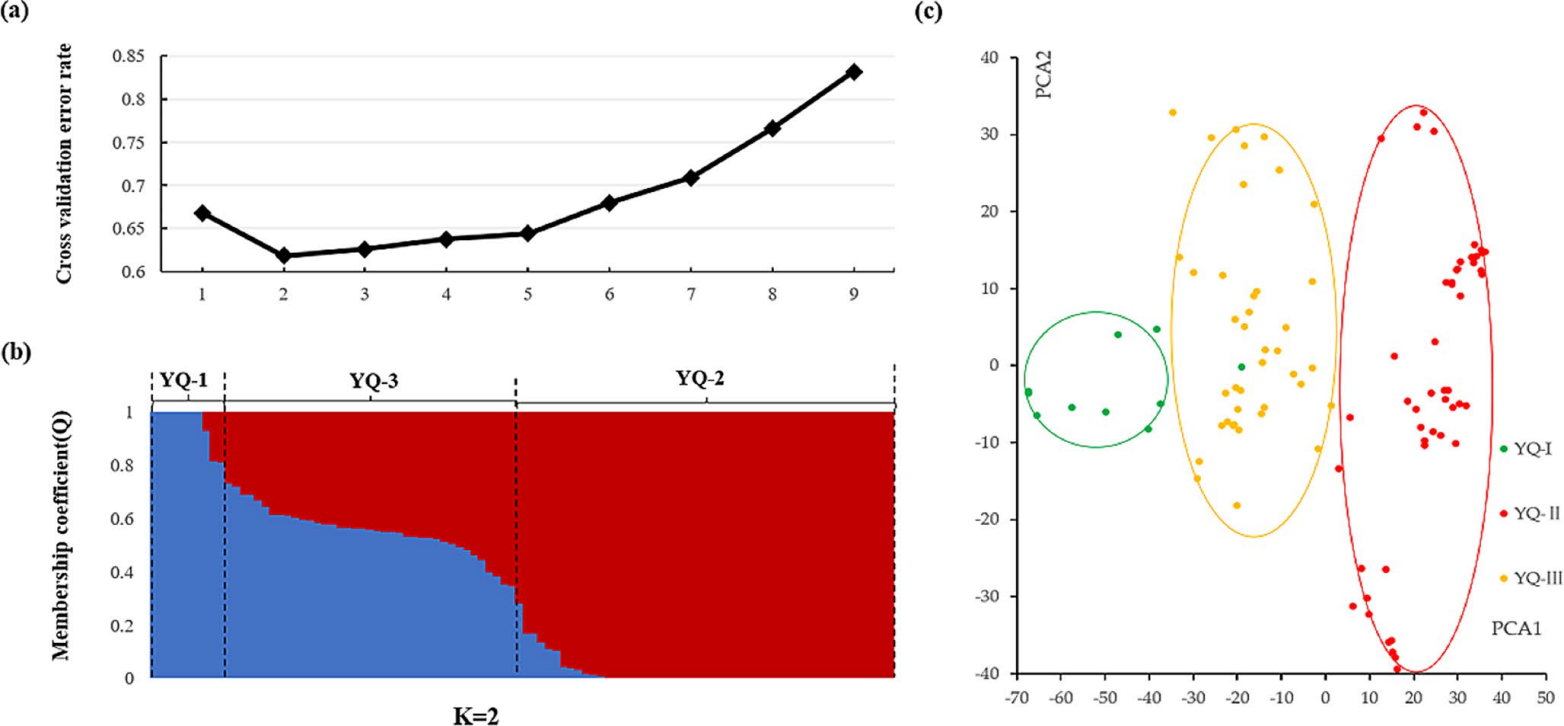
(**a**) Cross validation error rates corresponding to different k values (**b**) Inferred population structure of 100 accessions. Bar plot of individual membership coefficients for the genetic clusters inferred using ADMIXITURE (K = 2) base on 91,959 SNPs. Individual membership coefficients (Q) were sorted within each cluster. YQ-1 and YQ-2 are shown in blue and red, respectively (**c**) Principal component analysis (PCA). The three PCA scatter diagram was made by the first and second principal components

Figure 2 The distributions of SNPs on 15 chromosomes. The different color on each chromosome represented the number of SNPs within 1 Mb window size.

**Table 1:** Percentage of transformation and transversion SNPs identified using genotyping-by-sequencing

	Transformation			Transversion			
	AG	CT		AT	AC	CG	GT
Number of sites	266,725	267,010		46,046	36,680	26,492	37,986
Percentage	39.17%	39.21%		6.76%	5.39%	3.89%	5.58%
Total	78.38%			21.62%			

### Analysis of genetic distance and genetic diversity

Genetic distance is the basic parameter used in genetic diversity research, reflecting the phyletic evolution of groups. In this study, the genetic distance matrix between all 100 *C. tachangensis* accessions was calculated using MEGA software. Among the 4,950 combinations, the genetic distance ranged from 0.053 to 0.512, with an average of 0.26. The lowest genetic distance (0.053) was detected between accessions s394 and s397, both from Tongzi County (Northern Guizhou). The highest genetic distance (0.512) was found between accessions s279 and s348, from the Xingyi (Southwest Guizhou) and Sandu (Southern Guizhou) Counties of Guizhou province, respectively (Table S6). Among the 4,950 genetic distance combinations, 51.76%, 36.73%, and 6.79% were mainly distributed in the range of 0.250–0.350, 0.150–0.250, and 0.350–0.450 cM, respectively. Furthermore, 2.51% of the 4,950 genetic distance combinations were greater than 0.450 cM, and 2.20% were less than 0.150 cM (Fig. S2). In the genetic diversity analysis of *C. tachangensis* in Guizhou, we divided the 100 accessions into forest (62 accessions) and village (38 accessions) populations according to their growing area. The results show that the Tajima’s D values of the two populations were positive and the Ho, MAF, and Pi of the village population were 0.08, 0.14, and 0.22, respectively. The Ho, MAF, and Pi of the forest population were 0.09, 0.15 and 0.23, respectively. There was no significant difference in Fis between the forest and village populations (Table 2).

### Population structure analysis of *C. tachangensis* accessions in Guizhou Plateau

**Fig. 3:**
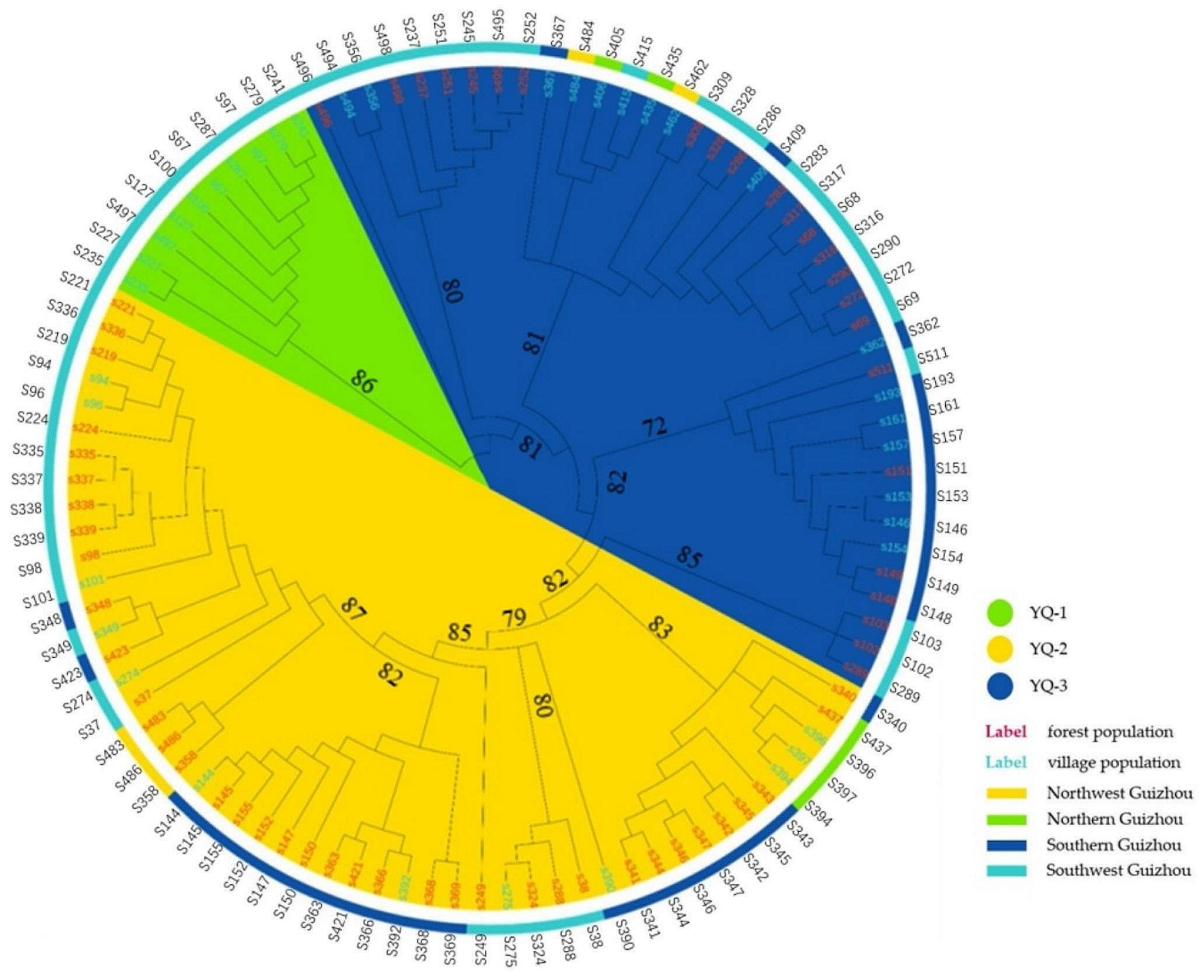
Phylogenetic analysis of 100 *C.tachangensis* accessions in Guizhou Plateau. Unroot Neighbor-Joining (NJ) phylogenetic tree was constructed using MEGA software with default parameter and used iTOL to visualize and color the tree. The different colored sector represents the different inferred populations, the leaf with different color represents the different population, and outer ring strip represents the different position

Changes in the population structure of 100 *C. tachangensis* accessions were further assessed under different K values based on 91,959 SNPs using ADMIXTURE software. Analysis of CV error revealed that K = 2 exhibited the minimum CV error. (Fig. 2a). The population structure at K = 2 was mainly clustered into three subgroups: YQ-1–3 (Fig. 2b). The membership function threshold was set to 0.8 and used to differentiate between pure and mixed subgroups. Thus, the 100 *C. tachangensis* accessions were divided into three subgroups: two pure subgroups (YQ-1 and YQ-2) and one mixed subgroup (YQ-3) (Fig. 2c). To investigate the phylogenetic relationships among the 100 *C. tachangensis* accessions, a phylogenetic tree was constructed based on the 91,959 SNPs using the neighbor-joining (NJ) method in MEGA software. We then used iTOL to visualize and colorize the obtained tree (Fig. 3). The YQ-1 subgroup contained 10 *C. tachangensis* accessions. Among them, 9(90%) grew in villages and 1(10%) in farmland, with all being distributed in Southwestern Guizhou. Thus, YQ-1 was designated the “Village Group”.The YQ-2 subgroup contained 50 accessions, composed of 38(76%) that grew in forests and 12(24%) that grew in villages. Of the former, 35(92%) grew in forests and 3(8%) grew near forests. Among the 50 accessions, 8% were distributed in Northern, 6% in Northwestern, 46% in Southern, and 40% in Southwestern GuizhouTherefore, YQ-2 was designated the “Forest Group”.

The YQ-3 subgroup contained 40 accessions, 24(60%) of which were distributed in the Forest Group and 16(40%) of which were distributed in the Village Groups. Among the former, 24(100%) grew near forests. Among the latter, 3(19%) grew in farmland and 13(81%) grew in villages. We regard the YQ-1 pure group from the region where were growing in village and the YQ-2 pure group from the region where were growing in forest, and the YQ-3 mixed group from the region where were growing near forest. Henceforth, YQ-3 was designated the “Transition Group.” Among its 40 accessions, 24(60%) were distributed in Southwestern, 30% in Southern, 5% in Northwestern, and 5% in Northern Guizhou.

### Analysis of genetic diversity and genetic composition of inferred population

The genetic diversity of three inferred populations was analyzed by Plink and VCFtools. The results show positive Tajima’s D values for the three inferred populations. The Ho, MAF, and Pi values of inferred population YQ-1 were 0.05, 0.11, and 0.16, respectively, being significantly lower than those of YQ-2, YQ-3, and the total population. The Ho, MAF, and Pi values of YQ-2 were 0.08, 0.14, and 0.20 (Table 2), representing significantly lower values than those of the total population and YQ-3. Genetic diversity parameters in YQ-3 were not significantly different from those in the total population. The three inferred populations did not differ significantly in terms of Fis.

**Table 2:** Genetic diversity analysis of *Camellia tachangensis* population

Group	Number	Ho	MAF	Pi	Fis	Tajima’s D
All	100	0.09a	0.15a	0.23a	0.65a	0.57
Forest	62	0.09a	0.15a	0.23a	0.62a	0.47
Village	38	0.08b	0.14b	0.22b	0.66a	0.46
YQ-1	10	0.05c	0.11c	0.16c	0.70a	0.55
YQ-2	50	0.08b	0.14b	0.20b	0.58a	0.58
YQ-3	40	0.09a	0.15a	0.22a	0.62a	0.47

*Note* *Ho* observed heterozygosity; *MAF* Minor Allele Frequency; *Pi* nucleotide diversity; *Fis* Inbreeding coefficient, significant difference analysis of abc representation

**Fig. 4:**
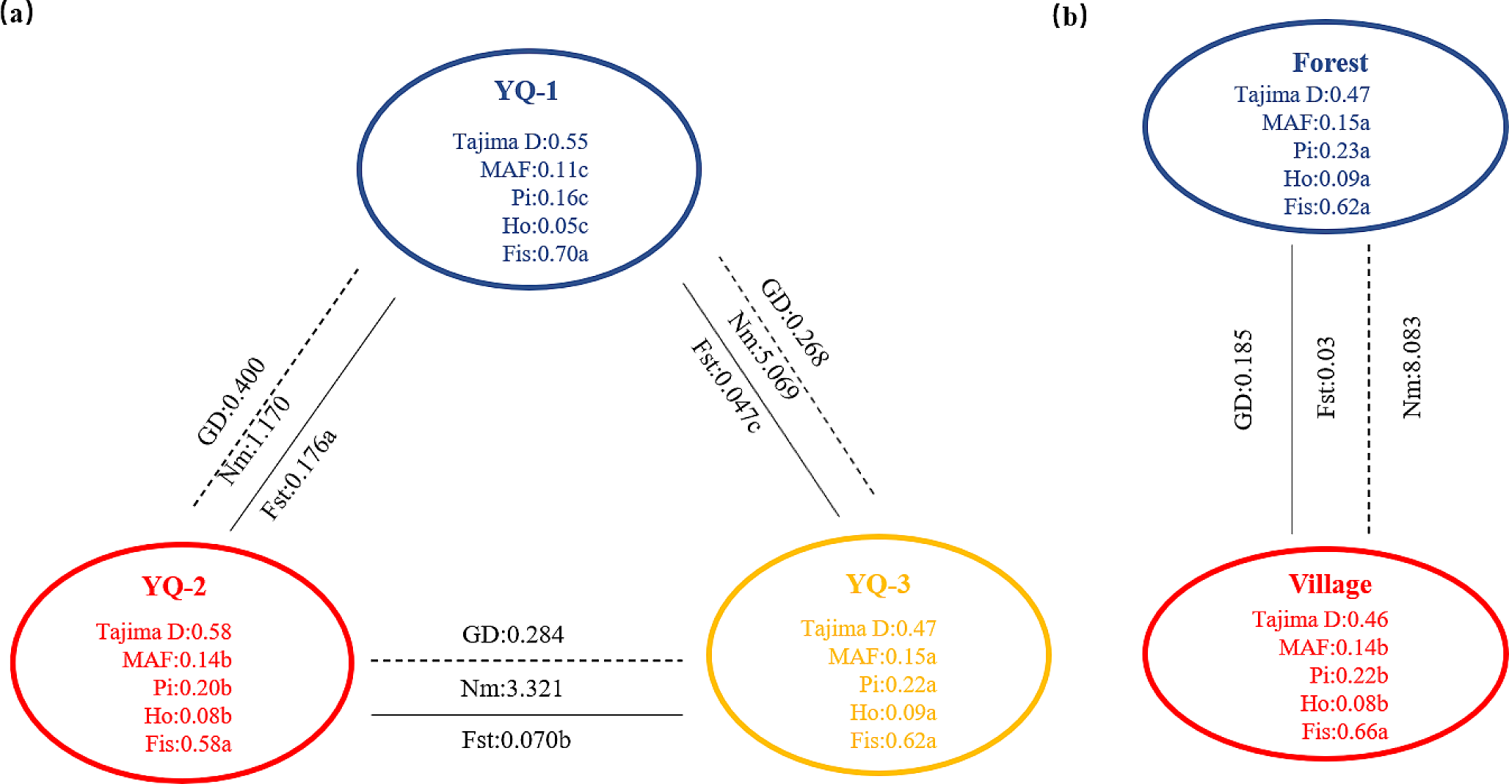
Genetic diversity of three inferred populations of 100 accessions. Pi nucleotide diversity, Ho observed heterozygosity, MAF minor allele frequency, Fis inbreeding coefficient, GD genetic distance, Fst differentiation coefficient. The different letters indicate a significant difference in *p* = 0.05 levels by the T-test. (**a**) YQ-1, YQ-2 are pure subgroups and the YQ-3 is the admixture subgroup base on ADMIXTURE software at K = 2 (**b**) forest population and village population base on accessions growth place

The Fst and genetic distance among the three inferred populations were analyzed by VCFtools and MEGA. The results revealed significant differences in Fst among the three inferred populations. The Fst and genetic distance between YQ-1 and YQ-2 were the largest, followed by those between YQ-2 and YQ-3, while those between YQ-1 and YQ-3 were the lowest. The Nm between YQ-1 and YQ-3 was the largest, while that between YQ-1 and YQ-2 was the smallest (Table S7, Fig. 4).

### LD analysis

**Fig. 5:**
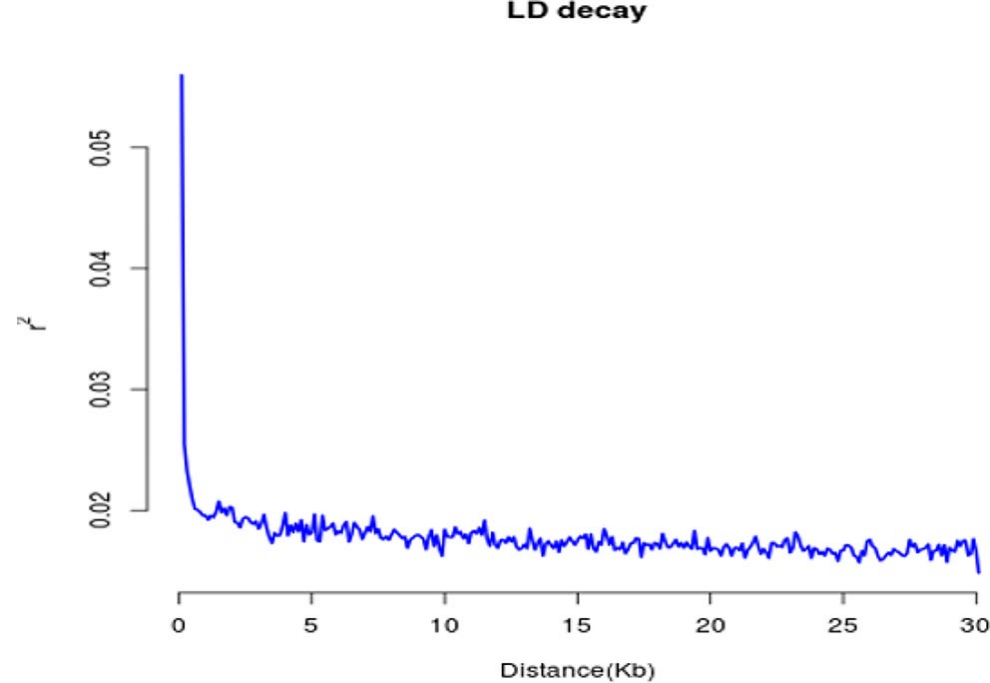
The Linkage disequilibrium decay plot of 100 *C. tachangensis* within 30KB. the x-axis represents the distance in KB and the y-axis represents the r^2^

LD analysis was used to explain domestication and mating history [6, 25]. In a population of 100 accessions, we used 29,393,327 non-LD-pruned SNPs to determine the level of LD. The LD decreased rapidly as genetic distance increased or with increasing distance between SNPs. The highest r^2^ values for LD decline among all 100 accessions were 0.25. As r^2^ decayed to half its maximum value (0.125), the equivalent physical distance was 0.2 Kb (Fig. 5, Table S8).

## Discussion

### Genetic distance and genetic diversity

Previous studies have demonstrated that the GBS method is effective for the genetic diversity analysis of tea plants [26, 27]. This study was the first to analyze the evolutionary relationships and genetic diversity of the *C. tachangensis* population. A total of 91,959 SNPs from 100 *C. tachangensis* accessions were analyzed using the GBS method. Genetic distance analysis was used to determine the genetic difference between a pair of specific accessions or populations, and usually shares many alleles with low genetic distance [28]. In the present study, the lowest genetic distance was beteween accessions from Northern Guizhou, belonging to YQ-2. The largest genetic distance was between accessions from Southwestern and Southern Guizhou, belonging to YQ-1 and YQ-2, respectively. The 10 pairs of accessions with minimum genetic distance were from the same inferred population and the same position. In 10 pairs of accessions with the larger pairwise genetic distance, there were 9 pairs of accessions from inferred population YQ-1 and YQ-2 respectively, and 2 pairs of accessions with the largest pairwise genetic distance from the different location, Southern and Southwest Guizhou respectively, which might be because *C. tachangensis* displays substantial genetic variation in the process of spreading to new areas.

The positive Tajima’s D values for all inferred populations suggested that they all underwent population bottlenecks and/or balancing selection. Moreover, the Ho, MAF, and Pi of the Forest Group were significantly higher than those of the Village Group. This indicates that the Village Group has lower genetic diversity due to the destruction of the surrounding natural environment and human selection, and that the Forest Group has not underwent excessive artificial selection and has retained high genetic diversity. The results of Zhao et al. (2014), who found that wild populations display greater diversity than recently domesticated populations, are similar to ours [29].

### Population structure

With the continuous development of high-throughput sequencing, the mining of SNP molecular markers through sequencing has been widely applied to tea plant kinship identification, population genetic diversity analyses [30], and genetic variation research [31]. Zhao et al. (2022) selected a total of 112,072 SNPs from the 253 tea accessions and performed a GBS analysis [32]. In our study, GBS technology was used to analyze the SNPs of 100 *C. tachangensis* accessions from the Guizhou Plateau. Three different methods were used to analyze their population structure based on the 91,959 high-quality SNPs, which further verified the accuracy of the population structure. Population genetic structure refers to a nonrandom distribution of genetic variation in species or populations [33, 34]. Through population structure analysis, the classification of relationships between individuals can be attained, populations can be divided into several inferred populations, and the occurrence of gene exchange between populations and the degree of hybridization of each individual can be elucidated [25, 33]. In this study, 100 *C. tachangensis* accessions were divided into three inferred populations according to their growth location: the Village Group (YQ-1), the Forest Group (YQ-2), and the Transition Group (YQ-3).

In this research, the genetic distance among the three inferred populations was consistent with the results of Fst and Nm. We observed the highest genetic differentiation and genetic distance and the lowest gene flow between Village and Forest Groups, the lowest genetic differentiation and genetic distance and the highest gene flow between Village and Transition Groups, and moderate genetic differentiation, genetic distance, and gene flow between Forest and Transition Groups. The lowest genetic diversity was detected in the Village Group, the highest was found in the Transition Group, and a moderate genetic diversity was detected in the Forest Group. These results support the notion that *C. tachangensis* evolution was related to human activity in the Guizhou Plateau. The Forest Group featured the most ancestral accessions originating in the forest without human intervention until present, and its retained group purity was owing to the limited pollen transmission distance in the forest, leading to plants that can only cross or self-cross within a small range. The Transition Group accessions were likely preserved because this plant is evergreen, grows slowly, and has no use value in the process of human long-term use of forests, deforestation and invasion of forests. Without the hindrance posed by forests, pollen among tea trees spreads farther, and tea trees with large genetic background differences were more likely to cross [35], which significantly increased the diversity of the Transition Group among all inferred groups. The Village Group underwent artificial selection because in our sampling process, we found that its accessions were located at the edge of terraces inside the village, playing the role of a retaining wall, or planted in front of villagers’ houses and used for brewing and drinking. In terms of evolutionary direction, the Forest Group was inclined to display high survival and the Village Group was inclined to produce higher yields and improved secondary metabolites for human consumption.The growth environments of the Village Group and Forest Group were separated by the Transition Group, which makes it difficult for them to have gene exchange. The reason for frequent gene exchange between the Village and Transition Groups might be that in recent years, with the expansion of human villages, the growth environment of the Transition Group was closer to that of the Village Group than that of the Forest Group. It might that village expanding into forest led the gene exchange between Forest Group and Village Group with increase of population and formed greater genetic diversity Transition Group. Overall, human activities have played an important role in the evolution of wild-type *C. tachangensis*. Although these accessions were not used for industrialization, a differentiation between *C. tachangensis* growing in villages and forests exists. Future research could focus on the protection of the Forest and Village Groups and the utilization of the Transition Group. The expansion of villages into forests may have led to gene exchange between Forest and Village Groups, leading to the formation of a Transition Group with a larger population and greater genetic diversity.

## Conclusions

In this study, 100 *C. tachangensis* accessions were collected, and SNP molecular data was used to perform a population structure analysis, which clustered them into three inferred populations: the Village Group (YQ-1), the Forest Group (YQ-2), and the Transition Group (YQ-3). The highest genetic differentiation and genetic distance and the lowest gene flow was between Village and Forest Groups, the lowest genetic differentiation and genetic distance and the highest gene flow was between Village and Transition Groups, and there was moderate genetic differentiation, genetic distance, and moderate gene flow between Forest and Transition Groups. The growth environment of the Village and Forest Groups was separated by the Transition Group, which decreased their gene exchange. The reason for the frequent gene exchange between the Village and Transition Groups might be that in recent years, with the expansion of human villages, the growth environment of the Transition Group was closer to that of the Village Group than that of the Forests Group. Additionally, the lowest genetic diversity was found in the Village Group, the highest genetic diversity was observed in the Transition Group, and a moderate genetic diversity was observed in the Forest Group. Without the hindrance of forests, pollen among tea trees spread farther and tea trees with large genetic background differences were more likely to cross, which significantly increased the diversity of the Transition Group among all inferred groups. These results support the notion that *C. tachangensis* evolution was related to human activity in the Guizhou Plateau. Future research could focus on the protection of Forest and Village Groups and the utilization of the Transition Group. The latter could play an important role in the future breeding of new varieties, and human activities will affect the genetic diversity of the *C. tachangensis* population.

## Electronic supplementary material

Below is the link to the electronic supplementary material.

Supplementary Material 1

Supplementary Material 2

Supplementary Material 3

## Data Availability

The raw sequence data reported in this study have been deposited in the Genome Sequence Archive in BIG Data Center, Beijing Institute of Genomics (BIG), Chinese Academy of Sciences, under accession number CRA001438 that is publicly accessible at Genome Sequence Archive (cncb.ac.cn).
