# Supplementary figures and images for: Analysis of population structure and genetic diversity of Camellia tachangensis in Guizhou based on SNP markers
Source: Mol Biol Rep. 2024 Jun 1;51(1):715. doi: 10.1007/s11033-024-09632-0 (PMC11144125; doi:10.1007/s11033-024-09632-0)

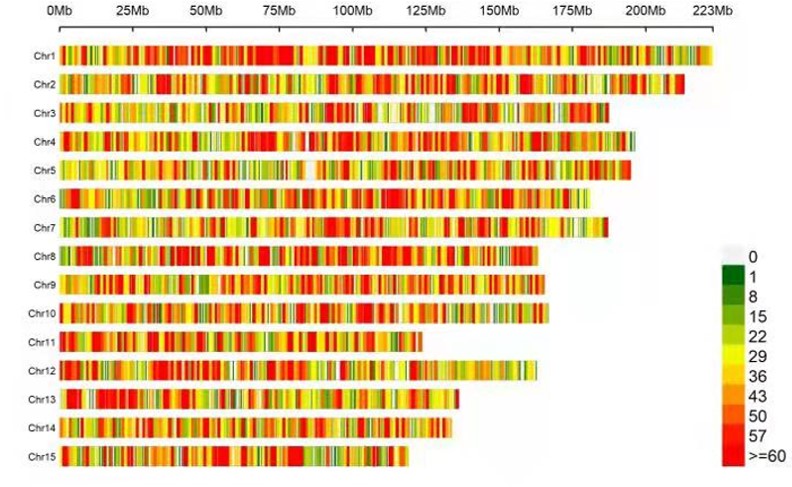

Supplement: Supplementary file 1 — Supplementary Material 1 [file 11033_2024_9632_MOESM1_ESM.jpg]

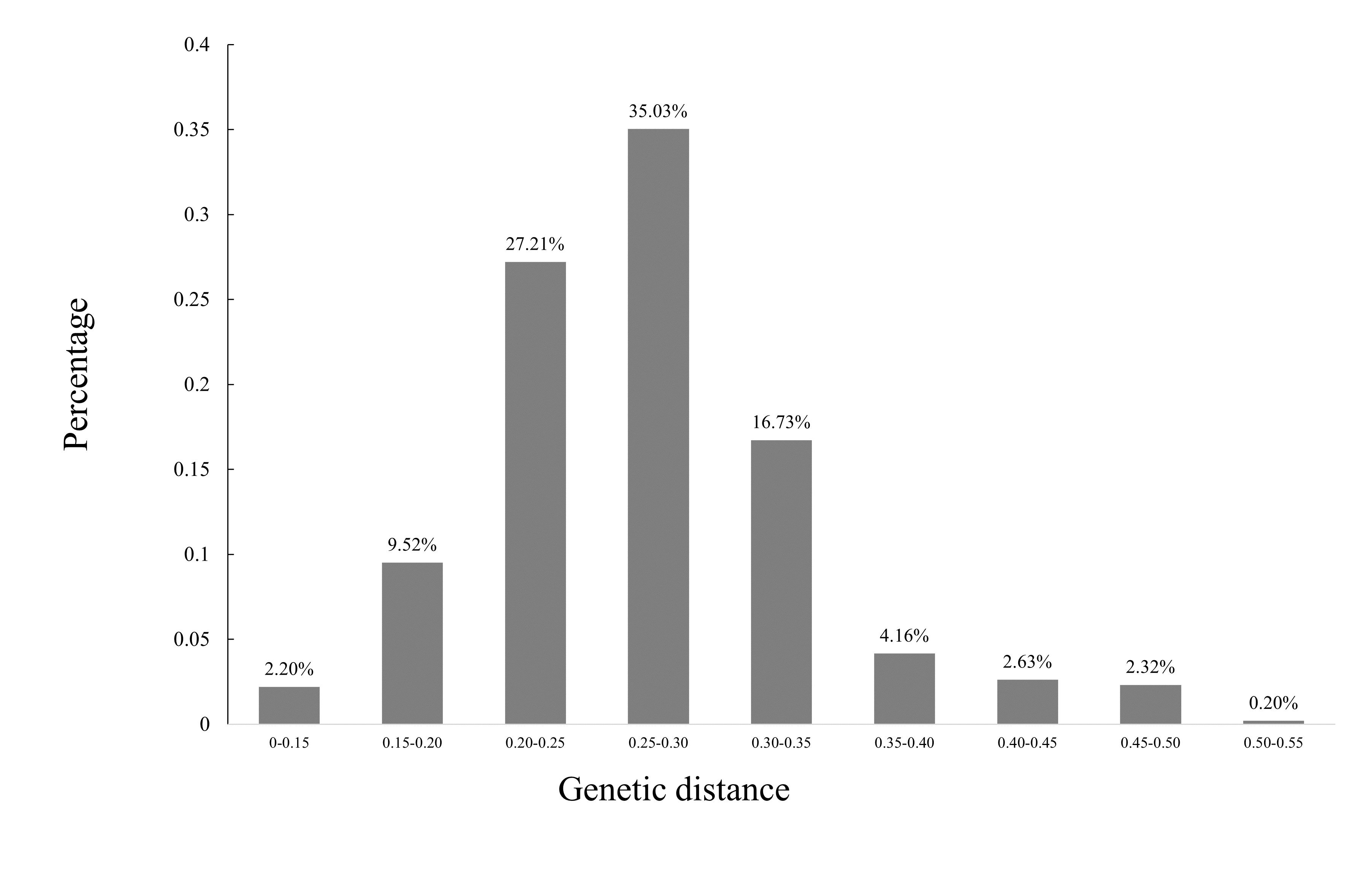

Supplement: Supplementary file 2 — Supplementary Material 2 [file 11033_2024_9632_MOESM2_ESM.png]
